# Qualidade da Anticoagulação Oral em Pacientes com Fibrilação Atrial em um Hospital Terciário no Brasil

**DOI:** 10.36660/abc.20210805

**Published:** 2022-06-23

**Authors:** Karina Nogueira Dias Secco Malagutte, Caroline Ferreira da Silva Mazeto Pupo da Silveira, Fabrício Moreira Reis, Daniele Andreza Antonelli Rossi, João Carlos Hueb, Katashi Okoshi, Hélio Rubens de Carvalho Nunes, Luis Cuadrado Martin, Rodrigo Bazan, Silméia Garcia Zanati Bazan

**Affiliations:** 1 Universidade Estadual Paulista Júlio de Mesquita Botucatu SP Brasil Universidade Estadual Paulista Júlio de Mesquita, Botucatu, SP – Brasil

**Keywords:** Fibrilação Atrial, Hemorragia, Varfarina, Acidente Vascular Cerebral

## Abstract

**Fundamento:**

A fibrilação atrial (FA) afeta de 0,5% a 2,0% da população geral e geralmente está associada a doenças estruturais cardíacas, comprometimento hemodinâmico e complicações tromboembólicas. A anticoagulação oral previne eventos tromboembólicos e é monitorada pela razão normalizada internacional (RNI).

**Objetivos:**

Avaliar a estabilidade do RNI em pacientes com FA não valvar tratados com anticoagulante varfarina, avaliar complicações tromboembólicas ou hemorrágicas e identificar o grupo com risco mais alto de eventos tromboembólicos ou hemorrágicos.

**Métodos:**

Dados de prontuários médicos de 203 pacientes atendidos em um hospital terciário no Brasil foram analisados e o tempo de intervalo terapêutico (TTR) foi calculado usando-se o método Rosendaal. Em seguida possíveis fatores que influenciam o TTR foram analisados e a relação entre TTR e eventos tromboembólicos ou hemorrágicos foi calculada. O nível de significância foi 5%.

**Resultados:**

O TTR médio foi 52,2%. Pacientes com instabilidade de RNI na fase de adaptação tinham um TTR médio mais baixo (46,8%) do que aqueles sem instabilidade (53,9%). Entre os pacientes estudados, 6,9% sofreram eventos hemorrágicos e 8,4% tiveram um acidente vascular cerebral. O grupo com risco mais alto de acidente vascular cerebral e sangramento era composto de pacientes com instabilidade de RNI na fase de adaptação.

**Conclusões:**

A qualidade da anticoagulação nesse hospital terciário no Brasil é semelhante à de centros de países em desenvolvimento. Pacientes com instabilidade de RNI maior na fase de adaptação evoluíram para um TTR médio mais baixo durante o acompanhamento, tinham uma chance de acidente vascular cerebral 4,94 vezes maior e uma chance de sangramento 3,35 vezes maior. Portanto, para esse grupo de pacientes, individualizar a escolha de tratamento anticoagulante seria recomendado, considerando-se a relação custo-benefício.

## Introdução

A fibrilação atrial (FA) é a arritmia cardíaca sustentada mais comum, afetando de 0,5% a 2,0% da população geral.^1 , 2^ Sua prevalência aumenta com a idade e é geralmente associada a doenças estruturais cardíacas, causando comprometimento hemodinâmico e complicações tromboembólicas com grandes implicações financeiras e um impacto significativo na morbidade e na mortalidade.^2 - 4^

O índice de acidente vascular cerebral em pacientes com FA é aproximadamente 5% por ano, que é de 5 a 7 vezes maior do que o de pacientes sem FA.^5^ Para evitar esses eventos embólicos cerebrais, a anticoagulação oral (ACO) é utilizada. A anticoagulação (com antagonistas da vitamina K (AVK), principalmente a varfarina) em pacientes com FA, independentemente da apresentação clínica, reduz a incidência de acidente vascular cerebral em aproximadamente 65 a 80%, diminuindo o risco anual de acidente vascular cerebral para 1,4% em comparação com o risco de 4,5% do placebo.^6 , 7^

A absorção, a farmacocinética e a farmacodinâmica da varfarina podem ser influenciadas por fatores genéticos, dieta e interações medicamentosas; esses fatores de influência conseguem potencializar ou diminuir o efeito anticoagulante. O objetivo da ACO é minimizar efetivamente o risco tromboembólico sem impacto significativo nos índices de hemorragia. Esse objetivo foi alcançado com uma razão normalizada internacional (RNI) de aproximadamente 2,5 (2,0-3,0)^8 , 9^ para pacientes com FA não valvar.

A anticoagulação por AVK demanda monitoramento constante pelo RNI, que se inicia já entre 5 e 7 dias após o início do tratamento e deve ser reavaliado a qualquer momento se houver alteração na dieta ou na dosagem de anticoagulante e/ou introduzir ou retirar outros medicamentos. A fase de adaptação à anticoagulação abrange os 6 primeiros meses de tratamento. Quando o RNI atinge a estabilidade, o monitoramento pode ser feito a cada 4 semanas.

A anticoagulação de longo prazo não é uma tarefa fácil e a aderência ao tratamento é essencial para evitar complicações tromboembólicas e hemorrágicas nos pacientes.

A baixa aderência dos pacientes a recomendações médicas e a baixa aderência dos médicos às diretrizes são desafios correntes para o tratamento por anticoagulação oral eficaz. A literatura mostra que não mais de 50% dos pacientes com recomendações de ACO recebem uma prescrição, e apenas 50 a 55% destes se encontram em uma faixa desejável de ACO, com 30 a 40% estando desprotegidos (RNI <2,0) e 10 a 15% ultrapassando o limite superior de RNI de 3,0.^10^

A ferramenta mais usada atualmente para avaliar a qualidade da anticoagulação em usuários de AVK é o cálculo do tempo de intervalo terapêutico (TTR).

Esse método, descrito por Rosendaal em 1993, usa uma interpolação linear para atribuir um valor de RNI a cada dia do intervalo entre as medições registradas.^11^

Estudos mostram que os valores de TTR abaixo de 60% estão relacionados a um risco maior de mortalidade global, sangramento importante, acidente vascular cerebral e tromboembolismo sistêmicos.^12^ No Brasil houve apenas alguns estudos empregando o método TTR para avaliar a qualidade da anticoagulação com AVK.

Esse estudo teve o objetivo de avaliar a estabilidade do RNI entre pacientes com FA não valvar e permanente, anticoagulados com AVK e que estão recebendo acompanhamento no ambulatório especializado de anticoagulação do Hospital das Clínicas da Faculdade de Medicina de Botucatu (HC-FMB-UNESP). O referido estudo também teve o objetivo de avaliar as complicações tromboembólicas e hemorrágicas nesses pacientes, como também de identificar o grupo em maior risco de eventos tromboembólicos ou hemorrágicos.

## Pacientes e métodos

Este é um estudo retrospectivo longitudinal em que foram incluídos 203 pacientes com FA não valvar permanente com mais de 18 anos de idade, que foram acompanhados por pelo menos 24 meses no ambulatório de anticoagulação do Hospital das Clínicas da Faculdade de Medicina de Botucatu (HC-FMB-UNESP) entre janeiro de 2009 e janeiro 2015. Pacientes que passaram mais de dois meses consecutivos sem consultas médicas no ambulatório foram excluídos.

Todos os procedimentos foram apresentados e aprovados pelo Comitê de Ética em Pesquisa (CEP) da Faculdade de Medicina de Botucatu (n° 445.651).

As variáveis clínicas e demográficas, a ocorrência de eventos tromboembólicos (acidente vascular isquêmico, ataque isquêmico transitório e êmbolos periféricos), e a ocorrência de eventos hemorrágicos importantes, tais como sangramento importante (que exige tratamento médico e/ou transfusão de sangue) e sangramento potencialmente fatal foram obtidas pela análise de registros médicos dos pacientes.

O TTR foi calculado para cada paciente dividindo-se o tempo em que o paciente permaneceu com um RNI dentro da faixa considerada aceitável (2,0 a 3,0) pelo tempo total de acompanhamento do paciente, e multiplicando-se o resultado dessa divisão por 100% para avaliar a qualidade da anticoagulação, e os fatores que poderiam influenciar o TTR. A relação entre TTR e a ocorrência de eventos tromboembólicos ou hemorrágicos também foram analisados.

### Análise estatística

As variáveis contínuas como distribuição normal e não normal são apresentadas como média e desvio padrão ou mediana e 25º e 75° percentis. A normalidade de variáveis numéricas foi avaliada usando-se o teste de Shapiro-Wilk. As variáveis categóricas são apresentadas como valores absolutos e porcentagens. O cálculo do valor de TTR seguiu o método descrito por Rosendaal em 1993. Portanto, o valor de TTR foi definido como: TTR = 100% (tempo total de acompanhamento com RNI entre 2 e 3) / tempo total de acompanhamento, o tempo total de acompanhamento com RNI entre 2 e 3 foi calculado obtendo-se o tempo entre duas medições de RNI (M1 e M2) e atribuindo metade do tempo para o valor de M1 e a outra metade do tempo ao valor M2, e assim por diante para todas as medições de RNI feitas para um determinado paciente. Ao final desse processo, é possível obter a soma total do tempo que um paciente passou com o RNI entre 2 e 3 dividir esse tempo pelo tempo total que esse paciente foi acompanhado.^11^ Vários modelos de regressão logística foram ajustados para explicar a chance de acidente vascular cerebral e sangramento em função do TTR e outras variáveis clínicas que foram estatisticamente significativas com p <0,20 nas associações bivariadas. No modelo de regressão múltipla final, as associações com p <0,05 foram consideradas significativas. A análise foi realizada com o software SPSS v21.0.

## Resultados

Um total de 203 pacientes com FA não valvar e permanente que foram acompanhados no ambulatório de anticoagulação de janeiro de 2009 a janeiro de 2015 (por um mínimo de 2 anos e um máximo de 10 anos) foram avaliados analisando-se seus prontuários médicos. As diretrizes do *American College of Chest Physicians* (Colégio Americano de Médicos do Peito)^13^ foram usadas para monitorar pacientes em terapia anticoagulante e os pacientes tiveram uma média de 43 consultas.

As variáveis clínicas e demográficas desses pacientes foram analisadas e são apresentadas na Tabela 1 .

**Tabela 1:** Características clínicas e demográficas de todos os pacientes (n=203 pacientes)

Variáveis	n	%
Idade (anos)	68 ± 9,7	
Não brancos	11	5,4
Pontuação CHA2DS2VASc	3 (3-4)	
Insuficiência cardíaca	78	38,4
Hipertensão	175	86,2
75 anos de idade ou mais	67	33,0
Diabetes mellitus	53	26,1
Acidente vascular cerebral ou AIT anterior	35	17,2
IM, PAo ou DAP	52	25,6
Entre 65 e 74 anos de idade	66	32,5
Masculino	114	56,2
Número de consultas	42 (26-63)	
HAS-BLED	2 (1-3)	
Sangramento anterior	2	1,0
Função renal alterada	22	10,8
Função hepática alterada	1	0,5
Alcoolismo	9	4,4
Hiperlipidemia	82	40,4
Tabagismo	67	33,0
Sedentarismo	132	65,0
Uso de antiplaquetários	26	12,8
Adaptação durante a instabilidade do RNI	48	23,6
TTR (%)	52 ± 17,2	
TTR abaixo de 60%	129	63,5
TTR abaixo de 65%	148	72,9
TTR abaixo de 70%	171	84,2
Acidente vascular cerebral durante a anticoagulação	17	8,4
Sangramento durante a anticoagulação	14	6,9
Acidente vascular cerebral ou sangramento durante a anticoagulação	30	14,8

*As variáveis contínuas são apresentadas como média ± desvio padrão, quando forem normalmente distribuídas, e como mediana e faixa interquartil (25%-75%) quando forem não normalmente distribuídas. As variáveis categóricas são apresentadas como valores absolutos e porcentagens. AIT: ataque isquêmico transitório; IM: infarto do miocárdio anterior; PAo: placa aórtica; DAP: doença arterial periférica; RNI: razão normalizada internacional; TTR: tempo de intervalo terapêutico.*

**Figura 1:**
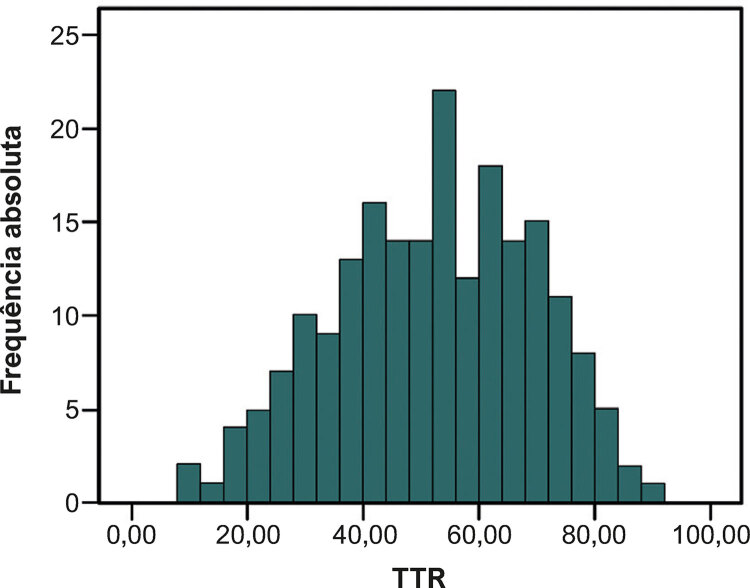
Histograma de valores de TTR.

Usando o método de interpolação linear proposto por Rosendaal, o TTR de cada paciente foi calculado, obtendo-se uma mediana de TTR de 53 (10-88) e uma média de 52,21% ( Figura 1 ).

**Figura 2:**
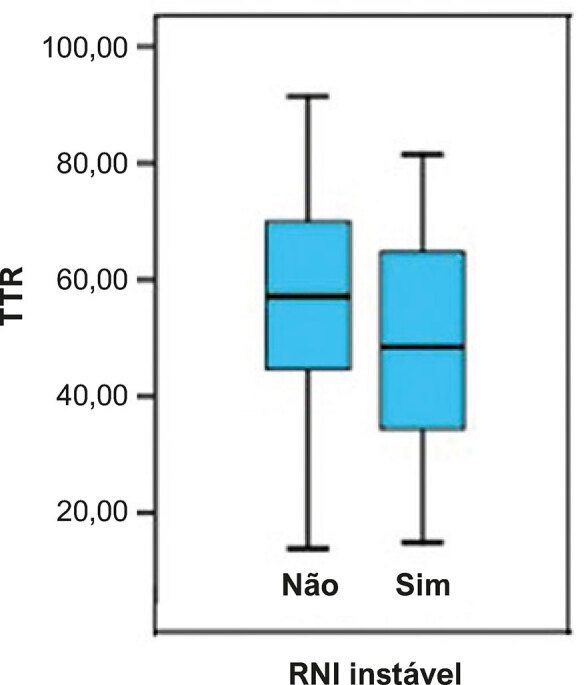
Diagrama de caixa de valores de TTR de acordo com a instabilidade durante a fase de adaptação.

Os fatores que influenciaram o valor de TTR dessa população foram analisados, e a instabilidade do RNI na fase de adaptação apresentaram uma relação inversa com o valor final de TTR. Pacientes que apresentaram RNI instável na fase de adaptação (RNI fora de nível terapêutico mais de 60% do tempo nos primeiros 6 meses de tratamento) tiveram o nível de TTR mais baixo (46,83%) que pacientes sem instabilidade (53,88%) ( Figura 2 ).

**Figura 3:**
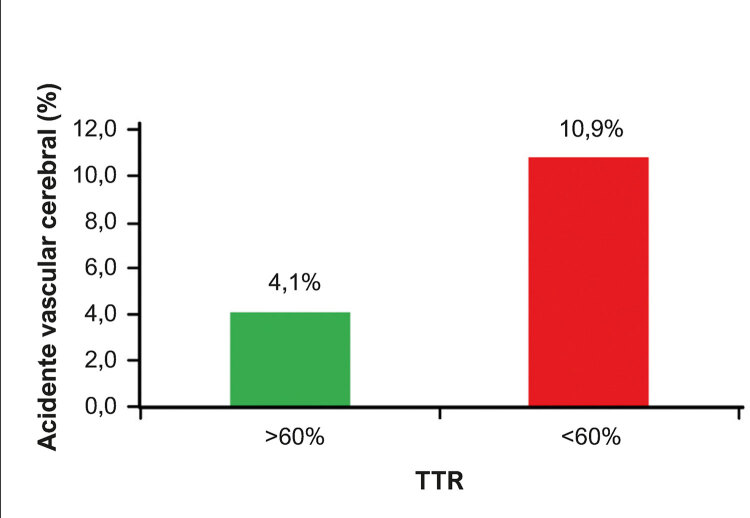
Porcentagem de pacientes com acidente vascular cerebral de acordo como valor de TTR durante o acompanhamento.

Entre os 203 pacientes estudados, 14 (6,9%) sofreram eventos hemorrágicos e 17 (8,4%) tiveram um acidente vascular isquêmico. Quando a relação entre a ocorrência de eventos importantes (acidente vascular cerebral e sangramento) e o valor de TTR foi analisada, concluiu-se que um TTR baixo (<60%) estava associado a uma maior ocorrência de acidente vascular cerebral ( Figura 3 ).

Outro fator associado à maior ocorrência de acidente vascular cerebral foi a instabilidade no RNI na fase de adaptação. Entre os pacientes com RNI instável durante o período de adaptação, o risco de acidente vascular cerebral era 4,94 vezes maior (RC=4,94 (1,62 – 15,02); p = 0,005) do que o risco para os pacientes sem instabilidade ( Tabelas 2 e 3 ).

**Tabela 2:** Regressão logística para risco de acidente vascular cerebral (associações bivariadas)

Variáveis	RC	IC 95%		p
Pontuação CHA2DS2VASc	1,29	0,92	1,81	0,135
Insuficiência cardíaca	1,13	0,41	3,11	0,808
Hipertensão	2,08	0,00	,	0,998
75 anos de idade ou mais	0,60	0,19	1,92	0,390
Diabetes mellitus	1,61	0,57	4,60	0,371
Acidente vascular cerebral ou AIT anterior	2,95	1,01	8,62	0,047
IM, PAo ou DAP	1,23	0,41	3,68	0,708
Entre 65 e 74 anos de idade	0,85	0,29	2,53	0,776
Masculino	0,67	0,25	1,82	0,432
HAS-BLED	1,31	0,79	2,18	0,288
Sangramento anterior	0,00	0,00	,	0,999
Função renal alterada	1,11	0,24	5,20	0,898
Função hepática alterada	0,00	0,00	,	1,000
Alcoolismo	1,39	0,16	11,83	0,763
Hiperlipidemia	0,79	0,28	2,23	0,655
Tabagismo	2,48	0,91	6,76	0,075
Sedentarismo	1,32	0,45	3,91	0,616
Uso de antiplaquetários	0,90	0,19	4,18	0,893
Adaptação durante a instabilidade do RNI	3,24	1,18	8,95	0,023
TTR	0,99	0,96	1,02	0,348
TTR abaixo de 60%	2,88	0,80	10,38	0,106
TTR abaixo de 65%	2,99	0,66	13,52	0,155
TTR abaixo de 70%	2,08	0,00	,	0,998

*AIT: ataque isquêmico transitório; IM: infarto do miocárdio anterior; PAo: placa aórtica; DAP: doença arterial periférica; RNI: razão normalizada internacional; TTR: tempo de intervalo terapêutico;*

**Tabela 3:** Regressão logística para risco de acidente vascular cerebral (modelo parcimonioso)

Variáveis	RC	IC 95%		p
Pontuação CHA2DS2VASc	1,62	1,04	2,53	0,031
Tabagismo	3,38	1,14	10,06	0,028
Adaptação durante a instabilidade do RNI	4,94	1,62	15,02	0,005

*RNI: razão normalizada internacional.*

Na análise dos fatores relacionados a sangramento, percebeu-se que pacientes com instabilidade de RNI durante a fase de adaptação tinham uma chance 3,35 maior de sangramento que os pacientes sem instabilidade ( Tabelas 4 e 5 ).

**Tabela 4:** Regressão logística para risco de sangramento (associações bivariadas)

Variáveis	RC	IC 95%		p
Pontuação CHA2DS2VASc	1,01	0,69	1,47	0,979
Insuficiência cardíaca	0,25	0,05	1,14	0,073
Hipertensão	2,17	0,27	17,25	0,465
75 anos de idade ou mais	1,14	0,37	3,54	0,823
Diabetes mellitus	2,27	0,75	6,87	0,148
Acidente vascular cerebral ou AIT anterior	0,35	0,04	2,77	0,321
IM, PAo ou DAP	0,78	0,21	2,91	0,711
Entre 65 e 74 anos de idade	1,61	0,54	4,85	0,395
Masculino	0,56	0,19	1,69	0,304
HAS-BLED	2,41	1,38	4,21	0,002
Sangramento anterior	14,46	0,86	244,62	0,064
Função renal alterada	3,80	1,08	13,36	0,037
Função hepática alterada	0,00	0,00	,	1,000
Alcoolismo	1,74	0,20	15,00	0,614
Hiperlipidemia	0,81	0,26	2,50	0,712
Tabagismo	1,57	0,52	4,74	0,420
Sedentarismo	3,45	0,75	15,87	0,112
Uso de antiplaquetários	1,15	0,24	5,44	0,864
Adaptação durante a instabilidade do RNI	3,61	1,20	10,88	0,023
TTR	1,01	0,98	1,04	0,642
TTR abaixo de 60%	0,75	0,25	2,25	0,607
TTR abaixo de 65%	0,65	0,21	2,03	0,455
TTR abaixo de 70%	1,13	0,24	5,32	0,875

*AIT: ataque isquêmico transitório; IM: infarto do miocárdio anterior; PAo: placa aórtica; DAP: doença arterial periférica; RNI: razão normalizada internacional; TTR: tempo de intervalo terapêutico.*

**Tabela 5:** Regressão logística para risco de sangramento (modelo parcimonioso)

Variáveis	RC	IC 95%		p
Diabetes mellitus	2,28	0,71	7,25	0,162
Função renal alterada	2,57	0,68	9,64	0,160
Adaptação durante a instabilidade do RNI	3,35	1,06	10,57	0,039

*RNI: razão normalizada internacional.*

## Discussão

Neste estudo, realizado em um hospital público, os TTR individuais foram calculados, e o valor médio foi 52,2%. Esse TTR médio é ligeiramente menor que o descrito em um hospital público, que apresentou um TTR médio de 56,6% (± 18,9).^14^ A literatura considera que níveis de TTR acima de 60% sejam indicativos de boa qualidade de coagulação,^15^ e, no presente estudo, apenas 36,5% se encontravam com um TTR acima de 60%. O estudo SPORTIF III e V,^12^ que incluiu 3587 pacientes, demonstrou que pacientes com um TTR abaixo de 60% apresentaram índices mais altos de mortalidade (4,20%) e sangramento importante (3,85%) quando comparados ao grupo com TTR entre 60% e 75% (1,84% e 1,96%, respectivamente) e ao grupo com TTR acima de 75% (1,69% e 1,58%, respectivamente). Embora esteja relacionado a uma ocorrência menor de eventos adversos, tais como sangramento e eventos tromboembólicos, um TTR acima de 60% não é alcançado facilmente em países em desenvolvimento como o Brasil. O estudo ROCKET AF^16^ realizado com 6983 pacientes de 1178 centros de 45 países demonstrou que o TTR, calculado de acordo com o método Rosendaal, varia de acordo com a região, com um TTR médio de 50,4% para pacientes do Leste Asiático, 35,9% para pacientes da Índia, 49,7% para pacientes do Leste Europeu, 54,8% para pacientes da África do Sul, 55,2% para pacientes da América Latina, 63,2% para pacientes da Europa Ocidental e 64,1% para pacientes do Canadá e dos Estados Unidos. Um TTR mais alto foi encontrado entre pacientes acompanhados em um ambulatório especializado em anticoagulação.^15 , 17^

No presente estudo, as características clínicas e demográficas de pacientes foram avaliadas, juntamente com o valor de TTR, e foi encontrada uma associação entre instabilidade de RNI na fase de adaptação à anticoagulação e um TTR mais baixo, o que significa que pacientes com valores de RNI instáveis durante a fase de adaptação apresentaram um TTR mais baixo (46,83%) durante todo o tratamento.

Esse estudo também estabeleceu a relação entre o TTR baixo e a ocorrência de um acidente vascular cerebral, demonstrando que quanto pior a qualidade da anticoagulação, maior a chance de acidente vascular cerebral. Os pacientes com um TTR médio abaixo de 60% apresentaram uma chance 2,88 maior de acidente vascular cerebral do que aqueles com um TTR médio acima de 60%. Outro achado desse estudo foi que pacientes que apresentaram RNI instável durante a fase de adaptação tinham um risco 4,94 vezes maior de acidente vascular cerebral e um risco 3,35 vezes maior de sangramento do que aqueles que não tinham instabilidade no RNI. Em relação à ocorrência de sangramento, não se encontrou uma relação estatisticamente significativa com um TTR médio baixo. Esse achado pode ser relacionado ao fato de que pacientes que mantiveram um TTR nesse estudo apresentaram principalmente medidas de RNI dentro da faixa terapêutica, e, portanto, com maior predisposição a acidente vascular cerebral do que a sangramento.

O valor médio de TTR e a ocorrência de eventos estão relacionados à aderência à terapia de anticoagulação e alguns fatores levam à não aderência aos AVK. A instabilidade de RNI, além de uma faixa terapêutica estreita, metabolismo variável, e possível dieta e interações medicamentosas, é uma limitação bem estabelecida do AVK. Esse fato levou ao surgimento de novas terapias de anticoagulação, e vários estudos importantes sobre anticoagulantes orais diretos (DOAC) foram publicados.^18 - 20^ Esses estudos têm um impacto semelhante na redução de eventos tromboembólicos em comparação com a varfarina, mas os DOAC apresentaram perfis de segurança semelhantes ou superiores. Além disso, à medida que os DOAC alcançam o início do efeito de anticoagulação mais rapidamente do que os AVK e sua ação é mais previsível, há menos necessidade de monitoramento terapêutico frequente, o que contribui para maior persistência com qualquer DOAC do que com os AVK, conforme observado por Ozaki et al.^21^

Embora o AVK tenha as limitações descritas anteriormente, o uso disseminado de DOAC em países em desenvolvimento é um desafio devido a limitações de custo, já que os custos são extremamente altos. Entretanto, vários estudos na Europa, nos Estados Unidos, no Canadá, na China e na África do Sul foram publicados para avaliar a relação custo-benefício, nos quais cada DOAC foi individualmente comparado à varfarina, e em todos eles ficou claro que o DOAC apresentou uma relação custo-benefício maior do que a varfarina.^22^

De acordo com um estudo realizado no Brasil, o custo mensal em dólares americanos por paciente fazendo a anticoagulação por varfarina é de $54,26, considerando as despesas com os profissionais de saúde envolvidos em consultas ambulatoriais para anticoagulação, custos laboratoriais para o monitoramento de RNI, aquisição de varfarina e custos indiretos, tais como dias de trabalho perdidos e transporte para a clínica. Os custos mensais médios de apixabana, dabigatrana e rivaroxabana para instituições públicas (de 1º de janeiro a 19 de agosto de 2015) foram $ 49,87, $ 51,40 e $ 52,16, respectivamente, demonstrando que os custos acumulativos para pacientes monitorados em um ambulatório de anticoagulação são mais altos para varfarina do que para os DOAC.^23^

Entretanto, ao avaliar exclusivamente os pacientes com FA, os custos do tratamento com varfarina eram semelhantes aos de tratamento com DOAC.^23^ Nesse caso, o conforto e a melhor aderência ao tratamento oferecido por um DOAC devido à não necessidade de monitorar o nível de anticoagulação, ao início e o fim rápidos do efeito de anticoagulação, à baixa interação medicamentosa, a ausência de interações com a dieta e, mais importante, a redução de eventos hemorrágicos cerebrais, devem ser levados em consideração, especialmente em alguns grupos de pacientes específicos, tais como aqueles com instabilidade de RNI durante a fase de adaptação, que provavelmente se beneficiariam da eficácia e da segurança de um DOAC.

### Limitações do estudo

As principais limitações do estudo são o tamanho da amostra, que pode ser pequeno para os fins do estudo, e o não tratamento de aspectos de aderência ao uso de AVK.

## Conclusão

Os resultados deste estudo nos permitem concluir que o TTR de pacientes acompanhados no ambulatório de anticoagulação do Hospital das Clínicas da Faculdade de Medicina de Botucatu (HC-FMB-UNESP), de janeiro de 2009 a janeiro de 2015, estavam abaixo daquilo descrito como ideal na literatura, como ocorre em outros países em desenvolvimento. Também se concluiu que a instabilidade do RNI na fase de adaptação foi um fator causal de TTR baixo e ocorrência mais alta de acidente vascular isquêmico e sangramento na população estudada.
