# Supplementary material for: Qualidade da Anticoagulação Oral em Pacientes com Fibrilação Atrial em um Hospital Terciário no Brasil
Source: Arq Bras Cardiol. 2022 Jun 23;119(3):363–9. [Article in Portuguese] doi: 10.36660/abc.20210805 (PMC9438533; doi:10.36660/abc.20210805)
Supplement: Translation

# Translation: Quality of Oral Anticoagulation in Atrial Fibrillation Patients at a Tertiary Hospital in Brazil

**Authors:** Karina Nogueira Dias Secco Malagutte, Caroline Ferreira da Silva Mazeto Pupo da Silveira, Fabrício Moreira Reis, Daniele Andreza Antonelli Rossi, João Carlos Hueb, Katashi Okoshi, Hélio Rubens de Carvalho Nunes, Luis Cuadrado Martin, Rodrigo Bazan, Silméia Garcia Zanati Bazan

**Affiliations:** 1 Brazil Universidade Estadual Paulista Júlio de Mesquita, Botucatu, SP – Brazil

**Keywords:** Atrial Fibrillation, Hemorrhage, Warfarin, Stroke

## Abstract

**Background:**

Atrial fibrillation (AF) affects 0.5% to 2.0% of the general population and is usually associated with cardiac structural diseases, hemodynamic damage, and thromboembolic complications. Oral anticoagulation prevents thromboembolic events and is monitored by the international normalized ratio (INR).

**Objectives:**

To evaluate INR stability in nonvalvular AF patients treated with warfarin anticoagulation, to evaluate thromboembolic or hemorrhagic complications, and to identify the group at higher risk for thromboembolic or hemorrhagic events.

**Methods:**

Data from the medical records of 203 patients who received medical care at a tertiary hospital in Brazil were reviewed, and the time in therapeutic range (TTR) was calculated using the Rosendaal method. The possible TTR influencing factors were then analyzed, and the relationship between the TTR and thromboembolic or hemorrhagic events was calculated. The level of significance was 5%.

**Results:**

The mean TTR was 52.2%. Patients with INR instability in the adaptation phase had a lower mean TTR (46.8%) than those without instability (53.9%). Among the studied patients, 6.9% suffered hemorrhagic events, and 8.4% had a stroke. The higher risk group for stroke and bleeding consisted of patients with INR instability in the adaptation phase.

**Conclusions:**

The quality of anticoagulation in this tertiary hospital in Brazil is similar to that in centers in developing countries. Patients with greater INR instability in the adaptation phase evolved to a lower mean TTR during follow-up, had a 4.94-fold greater chance of stroke, and had a 3.35-fold greater chance of bleeding. Thus, for this patient group, individualizing the choice of anticoagulation therapy would be advised, considering the cost-benefit ratio.

## Introduction

Atrial fibrillation (AF) is the most common sustained cardiac arrhythmia, affecting from 0.5% to 2.0% of the general population.^1 , 2^ Its prevalence increases with age, and it is generally associated with cardiac structural diseases, causing hemodynamic damage and thromboembolic complications with major economic implications and a significant impact on morbidity and mortality.^2 - 4^

The rate of stroke in nonvalvular AF patients is approximately 5% per year, which is 5 to 7-fold greater than that in patients without AF.^5^ To prevent such cerebral embolic events, oral anticoagulation (OAC) is employed. Anticoagulation (with vitamin K antagonists (VKA), notably warfarin) in AF patients, regardless of clinical presentation, reduces stroke incidence by approximately 65% to 80%, diminishing the annual risk of stroke to 1.4% versus the 4.5% risk with placebo.^6 , 7^

The absorption, pharmacokinetics, and pharmacodynamics of warfarin may be influenced by genetic factors, diet, and drug interactions; these influential factors are capable of potentializing or decreasing the anticoagulating effect. The OAC goal is to effectively minimize thromboembolic risk without a significant impact on hemorrhage rates. This goal was achieved with an international normalized ratio (INR) of approximately 2.5 (2.0-3.0)^8 , 9^ for nonvalvular AF patients.

VKA anticoagulation demands constant monitoring through the INR, which starts as early as 5 to 7 days after the onset of treatment and should be reevaluated at anytime if there is an alteration in diet or anticoagulant dosage and when introducing or withdrawing other drugs. The anticoagulation adaptation phase includes the first 6 months of treatment. After the INR reaches stability, monitoring may be carried out every 4 weeks.

Long-term anticoagulation is not an easy task, and adherence to treatment is paramount in order to avoid hemorrhagic and thromboembolic complications in patients.

Low patient adherence to doctor’s recommendations and poor doctor adherence to the guidelines are current challenges to effective oral anticoagulation treatment. The literature shows that no more than 50% of patients with OAC recommendations receive a prescription, and only 50-55% of those find themselves within the desirable range of OAC, with 30-40% unprotected (INR < 2.0) and 10-15% surpassing the upper INR limit of 3.0.^10^

The most employed tool currently used to evaluate anticoagulation quality in VKA users is calculating the time in therapeutic range (TTR).

This method, described by Rosendaal in 1993, uses a linear interpolation to assign an INR value to each day of the interval between the recorded measurements.^11^

Studies show that TTR values below 60% are related to a greater risk of death from all causes, major bleeding, stroke, and systemic thromboembolism.^12^ In Brazil, there have been only a few studies employing the TTR method to evaluate the anticoagulation quality with VKA.

This study aimed to evaluate INR stability among permanent and nonvalvular AF patients who were anticoagulated with VKA and who are currently undergoing follow-up in the specialized anticoagulation outpatient clinic of the Clinical Hospital of the School of Medicine of Botucatu (HC-FMB-UNESP). This study also aimed to evaluate the thromboembolic and hemorrhagic complications in these patients and to identify the group at greater risk for thromboembolic or hemorrhagic events.

## Patients and methods

This is a retrospective, longitudinal study in which 203 permanent and nonvalvular AF patients over 18 years of age, who had received follow-up for at least 24 months in the anticoagulation outpatient clinic of HC-FMB-UNESP between January 2009 and January 2015, were included. Patients who stayed for more than two consecutive months without doctor´s appointments in the outpatient clinic were excluded.

All procedures were submitted and approved by the Research Ethics Committee (CEP) of Botucatu Medical School (logged under protocol number 445.651).

The clinical and demographic variables, the occurrence of thromboembolic events (ischemic stroke, transitory ischemic accident, and peripheral emboli), and the occurrence of important hemorrhagic events, such as major bleeding (requiring medical treatment and/or blood transfusion) and life-threatening bleeding, were obtained through a review of patient medical records.

The TTR was calculated for each patient by dividing the time the patient remained with an INR within the range considered acceptable (2.0 to 3.0) by the patient’s total follow-up time and multiplying the result of this division by 100% in order to evaluate the anticoagulation quality and the factors that might influence TTR. The relation between TTR and the occurrence of hemorrhagic or thromboembolic events were also analyzed.

### Statistical analysis

Continuous variables with normal and non-normal distributions are presented as mean and standard deviation or median and^25th^and^75th^percentiles. The normality of numerical variables was assessed using the Shapiro-Wilk test. Categorical variables are presented as absolute values and percentages. The calculation of the TTR value followed the method described by Rosendaal in 1993. Thus, the TTR value was defined as: TTR= 100% x (total follow-up time with INR between 2 to 3) / total follow-up time, the total follow-up time with INR between 2 and 3 was calculated by having the time between two INR measurements (M1 and M2) and assigning one half of the time to the M1 value and the other half of the time to the value M2, and so on for all INR measurements made for a given patient. At the end of this process, it is possible to obtain the sum total of the time a patient spent with his INR between 2 and 3 and divided this time by the total time that this patient received follow-up.^11^ Multiple logistic regression models were adjusted to explain the chance of stroke and bleeding as a function of TTR and other clinical variables that were statistically significant with p < 0.20 in the bivariate associations. In the final multiple regression model, associations were considered significant when p < 0.05. The analysis was performed with SPSS v21.0 software.

## Results

A total of 203 patients with permanent and nonvalvular AF who were followed up in the anticoagulation outpatient clinic from January 2009 to January 2015 (for a minimum of 2 years and a maximum of 10 years) were evaluated through a review of their medical records. The guidelines of the American College of Chest Physicians^13^ were used to monitor patients with anticoagulant therapy, and the patients had an average of 43 outpatient visits.

Clinical and demographic variables from these patients were analyzed and are presented in Table 1 .

**Table 1:** Clinical and demographic characteristics of all patients (n=203 patients)

Variables	n	%
Age (years)	68 ± 9.7	
Nonwhite race	11	5.4
CHA2DS2VASc	3 (3-4)	
Heart failure	78	38.4
Hypertension	175	86.2
Age 75 years or older	67	33.0
Diabetes mellitus	53	26.1
Previous stroke or TIA	35	17.2
MI, AoP, or PAD	52	25.6
Age between 65 and 74 years	66	32.5
Male	114	56.2
Number of visits	42 (26-63)	
HAS-BLED	2 (1-3)	
Previous bleeding	2	1.0
Altered renal function	22	10.8
Altered liver function	1	0.5
Alcoholism	9	4.4
Hyperlipidemia	82	40.4
Smoking	67	33.0
Sedentary lifestyle	132	65.0
Antiplatelet use	26	12.8
INR instability during adaptation	48	23.6
TTR (%)	52 ± 17.2	
TTR under 60%	129	63.5
TTR under 65%	148	72.9
TTR under 70%	171	84.2
Stroke during anticoagulation	17	8.4
Bleeding during anticoagulation	14	6.9
Stroke or bleeding during anticoagulation	30	14.8

*Continuous variables are presented as mean ± standard deviation when normally distributed and median and interquartile range (25%-75%) when non-normally distributed. Categorical variables are presented in absolute values and percentages. TIA: transitory ischemic attack; MI: previous myocardial infarction; AoP: aortic plaque; PAD: peripheral artery disease; INR: international normalized ratio; TTR: time in therapeutic range.*

Using the linear interpolation method proposed by Rosendaal, the TTR of each patient was calculated, obtaining a median TTR of 53 (10-88) and a mean of 52.21% ( Figure 1 ).

**Figure 1:** Histogram of TTR values.

The factors that influenced the TTR value in this population were analyzed, and the instability of the INR in the adaptation phase presented an inverse relationship with the final value of the TTR. Patients who presented an unstable INR in the adaptation phase (INR out of therapeutic level more than 60% of the time in the first 6 months of treatment) had a lower mean TTR (46.83%) than patients without instability (53.88%) ( Figure 2 ).

**Figure 2:** Box-plot of TTR values according to instability during the adaptation phase.

Among the 203 studied patients, 14 (6.9%) suffered hemorrhagic events, and 17 (8.4%) suffered ischemic stroke. When the relationship between the occurrence of major events (stroke and bleeding) and TTR value was analyzed, it was concluded that a low TTR (<60%) was associated with a greater occurrence of stroke ( Figure 3 ).

**Figure 3:** Percentage of patients with stroke according to TTR value during follow-up.

Another factor associated with a greater occurrence of stroke was INR instability in the adaptation phase. Among patients with unstable INR during the adaptation period, the stroke risk was 4.94-fold greater (OR=4.94 (1.62 – 15.02); p = 0.005) than that in those without instability ( Tables 2 and 3 ).

**Table 2:** Logistic regression for stroke risk (bivariate associations)

Variables	OR	95% CI		p
CHA2DS2VASc	1.29	0.92	1.81	0.135
Heart failure	1.13	0.41	3.11	0.808
Hypertension	2.08	0.00	.	0.998
Age 75 years or older	0.60	0.19	1.92	0.390
Diabetes mellitus	1.61	0.57	4.60	0.371
Previous stroke or TIA	2.95	1.01	8.62	0.047
MI, AoP, or PAD	1.23	0.41	3.68	0.708
Age among 65 and 74 years	0.85	0.29	2.53	0.776
Male	0.67	0.25	1.82	0.432
HAS-BLED	1.31	0.79	2.18	0.288
Previous bleeding	0.00	0.00	.	0.999
Altered renal function	1.11	0.24	5.20	0.898
Altered liver function	0.00	0.00	.	1.000
Alcoholism	1.39	0.16	11.83	0.763
Hyperlipidemia	0.79	0.28	2.23	0.655
Smoking	2.48	0.91	6.76	0.075
Sedentary lifestyle	1.32	0.45	3.91	0.616
Antiplatelet use	0.90	0.19	4.18	0.893
INR instability during adaptation	3.24	1.18	8.95	0.023
TTR	0.99	0.96	1.02	0.348
TTR under 60%	2.88	0.80	10.38	0.106
TTR under 65%	2.99	0.66	13.52	0.155
TTR under 70%	2.08	0.00	.	0.998

*TIA: transitory ischemic accident; MI: previous myocardial infarction; AoP: aortic plaque; PAD: peripheral artery disease; INR: international normalized ratio; TTR: time in therapeutic range.*

**Table 3:** Logistic regression for stroke risk (parsimonious model)

Variable	OR	95% CI		p
CHA2DS2VASc	1.62	1.04	2.53	0.031
Smoking	3.38	1.14	10.06	0.028
INR instability during adaptation	4.94	1.62	15.02	0.005

*INR: international normalized ratio.*

In the analysis of the factors related to bleeding, it was perceived that patients with INR instability during the adaptation phase had a 3.35-fold greater chance of bleeding than those without instability ( Tables 4 and 5 ).

**Table 4:** Logistic regression for bleeding risk (bivariate associations)

Variables	OR	95% CI		p
CHA2DS2VASc	1.01	0.69	1.47	0.979
Heart failure	0.25	0.05	1.14	0.073
Hypertension	2.17	0.27	17.25	0.465
Age 75 years or older	1.14	0.37	3.54	0.823
Diabetes mellitus	2.27	0.75	6.87	0.148
Previous stroke or TIA	0.35	0.04	2.77	0.321
MI, AoP, or PAD	0.78	0.21	2.91	0.711
Age between 65 and 74 years	1.61	0.54	4.85	0.395
Male	0.56	0.19	1.69	0.304
HAS-BLED	2.41	1.38	4.21	0.002
Previous bleeding	14.46	0.86	244.62	0.064
Altered renal function	3.80	1.08	13.36	0.037
Altered liver function	0.00	0.00	.	1.000
Alcoholism	1.74	0.20	15.00	0.614
Hyperlipidemia	0.81	0.26	2.50	0.712
Smoking	1.57	0.52	4.74	0.420
Sedentary lifestyle	3.45	0.75	15.87	0.112
Antiplatelet use	1.15	0.24	5.44	0.864
INR instability during adaptation	3.61	1.20	10.88	0.023
TTR	1.01	0.98	1.04	0.642
TTR under 60%	0.75	0.25	2.25	0.607
TTR under 65%	0.65	0.21	2.03	0.455
TTR under 70%	1.13	0.24	5.32	0.875

*TIA: transitory ischemic accident; MI: previous myocardial infarction; AoP: aortic plaque; PAD: peripheral artery disease; INR: international normalized ratio; TTR: time in therapeutic range*

**Table 5:** Logistic regression for bleeding risk (parsimonious model)

Variables	OR	95% CI		p
Diabetes mellitus	2.28	0.71	7.25	0.162
Altered renal function	2.57	0.68	9.64	0.160
INR instability during adaptation	3.35	1.06	10.57	0.039

*INR: international normalized ratio.*

## Discussion

In this study, performed at a public hospital, the individual TTRs were calculated, and the mean value was 52.2%. This average TTR is slightly smaller than that described in a private hospital study, which presented a mean TTR of 56.6% (± 18,9).^14^ The literature considers TTR levels over 60% an indicator of good anticoagulation quality,^15^ and in the present study, only 36.5% of patients found themselves with a TTR over 60%. The SPORTIF III and V study,^12^ which included 3,587 patients, showed that patients with a TTR under 60% presented higher mortality (4.20%) and major bleeding (3.85%) rates when compared with the group with a TTR between 60% and 75% (1.84% and 1.96%, respectively), as well as with the group with a TTR over 75% (1.69% and 1.58%, respectively). Although it is related to a smaller occurrence of adverse events, such as bleeding or thromboembolic events, a TTR over 60% is not easily achieved in developing countries such as Brazil. The ROCKET AF study^16^ carried out with 6,983 patients from 1,178 centers from 45 countries demonstrated that the TTR, calculated according to the Rosendaal method, varies according to region, with a mean TTR of 50.4% for patients from East Asia, 35.9% for patients from India, 49.7% for patients from East Europe, 54.8% for patients from South Africa and 55.2% for patients from Latin America, 63.2% for patients from Western Europe, and 64.1% for patients from Canada/United States. A higher TTR was found among those patients followed up in a specialized anticoagulation outpatient care facility.^15 , 17^

In the present study, the clinical and demographic characteristics of patients were evaluated, along with the TTR value, and an association between INR instability in the anticoagulation adaptation phase and a lower TTR was found, meaning that patients with unstable INR values during the adaptation phase presented a lower TTR (46.83%) during the entire treatment.

This study also established a relationship between low TTR and the occurrence of stroke, showing that the worse the anticoagulation quality, the greater the chance of stroke. The patients with a mean TTR under 60% presented a 2.88-fold greater chance of stroke than those with a mean TTR over 60%. Another finding of this study was that patients who presented an unstable INR during the adaptation phase had a 4.94-fold greater risk of stroke and a 3.35-fold greater risk of bleeding than those who did not have INR instability.

Regarding the occurrence of bleeding, we did not find a statistically significant relationship with a low mean TTR. This finding may be related to the fact that patients who maintained a lower TTR in this study mainly presented INR measures below the therapeutic range and, therefore, with greater predisposition to stroke than bleeding.

The mean TTR value and the occurrence of events are related to the adherence to anticoagulation therapy, and some factors lead to a nonadherence to VKA. INR instability, in addition to a narrow therapeutic range, variable metabolism, and potential diet and drug interactions, is a well-established limitation of VKA. This fact pushed the emergence of new anticoagulation therapies, and several important studies on direct oral anticoagulants (DOAC) were published.^18 - 20^ These studies revealed a similar impact of reducing thromboembolic events when compared to warfarin, but the DOACs had similar or superior safety profiles. In addition, as DOACs reach the onset of an anticoagulation effect more quickly than AVK, and their actions are more predictable, there is less need for frequent therapeutic monitoring, which contributes to greater persistence with any DOAC than for VKA, as seen by Aya F. Ozaki et al.^21^

Although VKA has the previously described limitations, the disseminated use of DOAC in developing countries is challenged by cost limitations, as the costs are still extremely high. However, several studies in Europe, the United States, Canada, China, and South Africa were published to evaluate cost-effectiveness, in which each DOAC was individually compared with warfarin. In all of these, it was clear that the DOAC presented a greater cost-effectiveness than warfarin.^22^

According to a study carried out in Brazil, the monthly cost in dollar per patient who received anticoagulation with warfarin is $54.26, considering the expenses of health professionals involved in the anticoagulation outpatient visits, laboratory costs for INR monitoring, warfarin acquisition, and indirect costs, such as days of work missed and transportation to clinic. The mean monthly costs of apixaban, dabigatran, and rivaroxaban for public institutions (from January 1^st^to August 19^th^, 2015) were $49.87, $51.40, and $52.16, respectively, showing that the cumulative costs per patient followed up in an anticoagulation clinic are higher for warfarin than for DOACs.^23^

However, when exclusively evaluating AF patients, warfarin costs were similar to DOAC.^23^ In this case, the comfort and better adherence to treatment provided by a DOAC, since the patient does not need anticoagulation level monitoring, the fast onset and end of the anticoagulation effect, low drug interaction, absence of diet interaction, and, most importantly, the reduction in cerebral hemorrhagic events should be taken into account, especially in some specific patient groups, such as those with INR instability during the adaptation phase, which would most likely benefit from the efficacy and safety of a DOAC.

### Study limitations

The main limitations of this study are the sample size, which may be small for the purposes of the study, and the failure to address aspects of adherence to the use of VKA.

## Conclusion

The results of this study allow us to conclude that the TTR of patients who received follow-up at the anticoagulation outpatient clinic of the Clinical Hospital of the School of Medicine of Botucatu (HC-FMB-UNESP), from January 2009 to January 2015, was below what is described as ideal in the literature, as occurs in other developing countries. It can also be concluded that the instability of the INR in the adaptation phase was a causal factor for both a low TTR and a higher occurrence of ischemic stroke and bleeding in the population studied.
